# Editorial: Pediatric respiratory critical illness: etiology, diagnosis, and treatment

**DOI:** 10.3389/fped.2025.1677006

**Published:** 2025-08-14

**Authors:** Hongxing Dang, Rujipat Samransamruajkit, Karen Ka Yan Leung

**Affiliations:** ^1^ Children’s Hospital of Chongqing Medical University, Chongqing, China; ^2^ Faculty of Medicine, Chulalongkorn University, Bangkok, Thailand; ^3^ Hong Kong Children’s Hospital, Kowloon, Hong Kong SAR, China

**Keywords:** respiratory insufficiency, bronchopulmonary dysplasia, mechanical ventilation, biomarkers, pediatric intensive care units

**Editorial on the Research Topic**
Pediatric respiratory critical illness: etiology, diagnosis, and treatment

## Introduction

Severe respiratory diseases—including acute respiratory distress syndrome (ARDS), bronchopulmonary dysplasia (BPD), and severe pneumonia—remain leading causes of pediatric morbidity and mortality worldwide. Despite decades of advances, mortality from pediatric ARDS remains high, particularly in patients with sepsis or multi-organ dysfunction (1, 2). BPD affects more than 20% of extremely low birth weight infants and contributes to prolonged hospital stays and long-term respiratory complications (3). Globally, pneumonia remains a top cause of death among children under (4). These challenges highlight the urgent need for improved diagnostics, monitoring tools, and targeted therapies.

This Research Topic gathers nine original contributions that offer valuable insights into early assessment, physiologic monitoring, clinical management, and rare disease phenotypes in pediatric respiratory critical illness.

## Innovations in early assessment and risk stratification

Qin et al. explored the use of exhaled nitric oxide (FeNO) and tidal breathing parameters to assess airway hyperresponsiveness (AHR) in infants under 3 years of age. Their findings reinforce the predictive value of FeNO >14 ppb and flow-volume ratios in suspected asthma—a significant advance for a difficult-to-test age group. This aligns with recent pediatric studies showing FeNO as a noninvasive surrogate for eosinophilic inflammation and long-term asthma risk (5).

Colak et al. introduced renal near-infrared spectroscopy (RrSO₂) as a physiologic marker for extubation failure in ventilated children. A >6.15% drop in RrSO₂ during readiness testing had a sensitivity of 98.4% and specificity of 88.9%, suggesting it may be a reliable adjunct to respiratory monitoring in complex cases.

## Respiratory support and weaning models

Ge et al. developed a multifactorial prediction model integrating P/F ratio, diaphragm ultrasound indices (DE-RSBI, DTF-RSBI), and Pediatric Critical Illness Score (PCIS). This model significantly outperformed individual variables (AUC = 0.96) in predicting successful weaning. The study supports a shift from single-parameter thresholds to multimodal, data-driven decision tools.

Banik et al. evaluated the usability of the Vayu bCPAP system in five neonatal care facilities in Bangladesh. Providers reported strong acceptability and effectiveness in treating respiratory distress syndrome (RDS), highlighting how low-cost, portable innovations can improve neonatal care in resource-limited settings.

## Mechanistic insights and biomarker discovery

Li et al. reviewed the role of oxidative stress in the pathogenesis of BPD. Their analysis identified 8-hydroxy-2'-deoxyguanosine (8-OHdG) as a promising biomarker for early detection, adding to a growing body of evidence supporting biomarker-guided neonatal care (5).

Duenas-Meza et al. studied sleep-disordered breathing in children with cystic fibrosis at high altitude. They found that total sleep time with oxygen saturation <90% and <85% correlated negatively with FEV₁, reinforcing the importance of polysomnography in evaluating pulmonary function in high-altitude environments.

### Case-based contributions: rare and complex presentations

Zhou et al. assessed bronchoalveolar lavage (BAL) in treating small airway diseases. Compared with conventional therapy, BAL accelerated symptom resolution, imaging recovery, and reduced re-hospitalization, suggesting a potential benefit in patients with recurrent or persistent lower airway disease.

Liu et al. described two Chinese siblings with FINCA syndrome caused by a novel NHLRC2 mutation. One child responded to long-term corticosteroids, indicating that anti-inflammatory therapy may offer benefit in genetically mediated interstitial lung disease.

Yang et al. presented a case of *Mycoplasma pneumoniae*-induced diffuse alveolar hemorrhage (DAH) complicated by hemophagocytic lymphohistiocytosis (HLH). The case demonstrated the importance of metagenomic next-generation sequencing (mNGS) and early recognition of HLH in life-threatening infections.

## Conclusion

Together, these articles underscore critical advancements in pediatric respiratory critical care: Noninvasive biomarkers such as FeNO and RrSO₂ offer new pathways for early detection and monitoring; Multimodal prediction models outperform traditional approaches in ventilator weaning; Low-cost devices like Vayu bCPAP expand respiratory care access in LMICs; Precision recognition of rare and complex conditions facilitates timely and targeted interventions.

This Research Topic highlights how integrated, multidisciplinary efforts are shaping the future of pediatric respiratory care. We thank all contributing authors and reviewers for their valuable insights.
